# Factors influencing an eruption of teeth associated with a dentigerous cyst: a systematic review and meta-analysis

**DOI:** 10.1186/s12903-021-01542-y

**Published:** 2021-04-07

**Authors:** Marek Nahajowski, Sylwia Hnitecka, Joanna Antoszewska-Smith, Kornelia Rumin, Magdalena Dubowik, Michał Sarul

**Affiliations:** 1 Department of Dentofacial Orthopedics and Orthodontics, Wroclaw Medical University, Krakowska 26, 50-425 Wrocław, Poland; 2 Department of Maxillofacial Surgery, Wroclaw Medical University, Borowska 213, 50-556 Wrocław, Poland

**Keywords:** Systematic review, Dentigerous cyst, Eruption interval, Orthodontics

## Abstract

**Background:**

A dentigerous cyst (DC) is a pathology embracing the crown of an unerupted tooth at risk of malignant transformation. The causal tooth is usually removed together with the cyst. However, if there are orthodontic contraindications for extraction, two questions arise. (1) Which factors favor spontaneous eruption? (2) Which factors imply the necessity of applying orthodontic traction? This systematic review aimed to identify factors conducive/inconducive to the spontaneous eruption of teeth after dentigerous cyst marsupialization.

**Methods:**

In accordance with the PRISMA guidelines, the main research question was defined in the PICO format (P: patients with dentigerous cysts; I: spontaneous tooth eruption after surgical DC treatment; C: lack of a spontaneous tooth eruption after surgical DC treatment; O: determining factors potentially influencing spontaneous tooth eruption). The MEDLINE, EMBASE, and Cochrane Central Register of Controlled Trials databases were searched for keywords combining dentigerous/odontogenic/follicular cysts with teeth and/or orthodontics, as well as human teeth and eruption patterns/intervals/periods/durations. The following data were extracted from the qualified articles (4 out of 3005 found initially): the rate of tooth eruption after surgical treatment of the cyst, the age and sex of the patients, the perpendicular projection distance between the top of the tooth cusp and the edge of the alveolar process, tooth angulation, the root formation stage, the cyst area, and the eruption space. The articles were subjected to risk of bias and quality analyses with the ROBINS-I protocol and the modified Newcastle–Ottawa QAS, respectively. Meta-analyses were performed with both fixed and random effects models. The GRADE approach was used to evaluate the quality of the evidence. The systematic review was registered in PROSPERO under ID CRD42020189044.

**Results:**

Nearly 62% of DC-associated premolars erupted spontaneously after cyst marsupialization/decompression. Young age (mean = 10 years) and root formation not exceeding 1/2 of its fully developed length were the factors likely to favor spontaneous eruption.

**Conclusion:**

The small number of published studies, as well as their heterogeneity and the critical risk of bias, did not allow the creation of evidence-based protocols for managing teeth with DC after marsupialization. More high-quality research is needed to draw more reliable conclusions.

**Supplementary Information:**

The online version contains supplementary material available at 10.1186/s12903-021-01542-y.

## Background

According to the definition established by the World Health Organization, a dentigerous cyst (DC), formerly known as a follicular cyst, is a pathology embracing the crown of an unerupted tooth that attaches to its cementoenamel junction [1, 2]. Such cysts sometimes transform into neoplastic compositions, which has been presented in several case reports [3, 4]. DCs are the second most common bone cyst and affect 0.91–7.3% of the population [5, 6]. They are often located near the lower third molars and near the upper canines, lower premolars, and upper third molars. DCs lead to a lack of teeth in the oral cavity or a delay in eruption, often complicated by an incorrect tooth position in the dental arch [7, 8].

For this reason, in addition to the decompression/enucleation of the cyst, it is essential to provide a proper intervention for teeth whose eruption process has been disturbed. The wisdom teeth are usually removed together with the cyst. However, other teeth—mainly canines in the maxilla and premolars in the mandible—should be maintained to secure continuity of the dental arches [8–12]. In such cases, the literature provides two options: (1) observation of the tooth after surgical intervention, waiting for its spontaneous eruption, as well as the implementation of orthodontic treatment only if this process is delayed; or (2) immediate loading of the tooth with orthodontic force [13–21]. Regardless of many factors potentially influencing a spontaneous eruption, thus forcing the choice of one or the other treatment protocol [15, 19, 22–25], this issue has not been statistically assessed in a systematic review so far.

The study aimed to determine whether an evidence-based protocol for the clinical management of DC-associated teeth based on the factors provided in the literature that indicate whether the tooth would spontaneously erupt after the surgical treatment of a cyst can be established.

## Methods

The systematic review was registered in PROSPERO under ID CRD42020189044. The study was performed according to the PRISMA (Preferred Reporting Items for Systematic Reviews and Meta-Analyses) guidelines, and the main research question was defined in the PICOS (Population, Intervention, Comparison, Outcome, Study design) format (Table 1).

**Table 1:** PICOS format

Parameter	Inclusion criteria
Population	Patients of any age, gender and ethnicity with the teeth associated with dentigerous cysts
Intervention	Spontaneous tooth eruption after surgical DC treatment
Comparison	Lack of a spontaneous tooth eruption after surgical DC treatment
Outcome	Time to eruption; factors potentially influencing spontaneous tooth eruption, e.g. root formation stage, tooth angulation, distance between the top of a tooth cusp and the edge of the alveolar process, cyst area, eruption space, age, gender
Study design	Randomized clinical trials (RCT), prospective controlled clinical trials (CCT), case series, observational studies, review articles, and retrospective studies; published in English

The following electronic databases were searched: PubMed, EMBASE, and the Cochrane Central Register of Controlled Trials. The search strategy is shown in Additional file 1.

Based on information from the titles and abstracts, relevant articles meeting the following inclusion criteria were selected: randomized clinical trials (RCTs), prospective controlled clinical trials (CCTs), case series, observational studies, review articles, and retrospective studies of generally healthy patients with DC-associated teeth. Single case reports, studies with limited data including conference abstracts and journal letters, studies of nonrepresentative groups, studies of patients with syndromes, and animal studies were excluded. Among the selected full-text articles, those written in a language other than English, those not related to the current systematic review subject and those with a sample size of less than 5 per group were excluded. The bibliographies of the remaining articles were reviewed. The following journals were manually screened: the American Journal of Orthodontics and Dentofacial Orthopedics, International Orthodontics, the Journal of Clinical Orthodontics, the Journal of Oral and Maxillofacial Surgery, the Journal of Maxillofacial and Oral Surgery, the Journal of Stomatology, Oral and Maxillofacial Surgery, BMC Oral Health, the Journal of Clinical Pediatric Dentistry, the Journal of Clinical and Experimental Dentistry, and the Journal of Dentistry for Children. The literature search, assessment of relevance, risk of bias analysis, and data extraction were performed independently by two authors (MN and SH). All authors discussed disagreements until consensus was reached.

The following data were extracted from the included studies: sample size, year of publication, time elapsed from surgical treatment of the cyst to tooth eruption, and data potentially affecting the eruption process, such as patient age and sex, root formation stage, perpendicularly measured distance from the alveolar ridge to the top of the tooth cusp, tooth angulation, cyst area, and eruption space.

An analysis of the risk of bias in individual articles was performed using the ROBINS-I (Risk of Bias In Nonrandomized Studies of Interventions) tool, assessing the following domains: (1) pre-intervention domain (bias due to confounding and bias in the selection of participants into the study), (2) at-intervention domain (bias in the classification of interventions) and (3) post-intervention domain (bias due to deviations from intended interventions, bias due to missing data, bias in the measurement of outcomes and bias in the selection of the reported results). The risk of bias in each of the articles was determined based on the lowest score awarded in all criteria. The risk of bias in all four studies was assessed as critical.

For a qualitative assessment, a modified Newcastle–Ottawa Quality Assessment Scale (QAS) was used to analyze the following:

1. Patient selection: A. representativeness of the group exposed to the tested factor, B. selection of patients for the control group, C. providing the source of data for individual patients, and D. demonstrating that the effects did not occur at the beginning of the study. For each positively assessed aspect, a maximum of 1 point was awarded.
2. The presence of confounding factors causing the control group to not affect the results in the same way as the study group. In this category, 0 to 2 points were awarded, depending on the significance of the influence of confounding factors.
3. Outcomes affected by: (A) the blinding of the evaluators, (B) a follow-up duration of longer than 12 months, and (C() more than 75% of patients who completed the study, which allowed for a maximum of 3 points.

Random-effects meta-analyses of the risk ratios of spontaneous eruption and (a) the stage of root formation together with the participants’ mean differences in age, (b) cusp depth, (c) bud angulation and (d) eruption space were carried out, while the mean differences in the cyst area were assessed by a fixed effects meta-analysis. The significance was established at *p* < 0.1. The Cochrane Q test and I^2^ statistics enabled the evaluation of statistical heterogeneity of the collected data. Studies with an I^2^ statistic of more than 50% were considered to have high heterogeneity. All calculations were performed with the Statistica 13 PL software (StatSoft Polska, Krakow, Poland). The GRADE (Grading of Recommendations Assessment, Development, and Evaluation) approach was used to assess the quality of evidence.

## Results

**Fig. 1:**
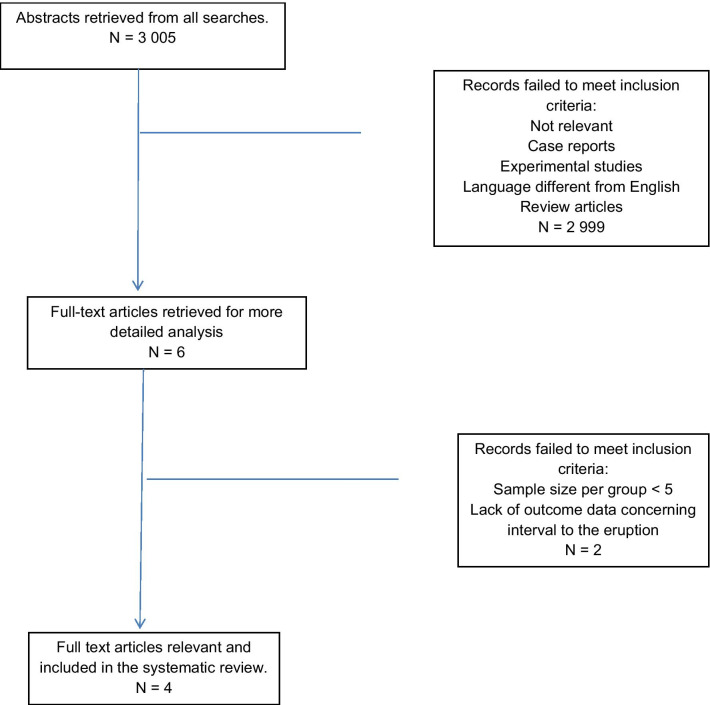
Flow chart

With the abovementioned keywords, 3005 abstracts were retrieved. No articles describing RCT or CCT results were found, nor were there any literature reviews on the tooth eruption rate. There were only a few clinical studies and articles on the patients’ mean age at the time of the eruption of subsequent teeth, without specifying the duration of the process. Six papers were qualified for inclusion in the systematic review, two of which were ultimately excluded. The complete selection process is shown in Fig. 1.

A direct comparison of the results was possible because all the studies focused on DC-associated lower premolars.

All selected articles were single-center retrospective cohort studies using the patients’ clinical data and measurements taken from panoramic radiographs. In all papers, the patients were allocated into groups (study or control) after the completion of treatment. Fuji et al. [22] analyzed a group of 60 patients divided into two subgroups: (1) patients with spontaneous tooth eruption after cyst marsupialization (45 individuals) and (2) patients treated with cystectomy or orthodontic traction (15 individuals). In the study by Hyomoto et al. [23], there were 47 patients with DC-associated premolars in the mandible and 11 patients with DC-associated canines in the maxilla. To maintain homogeneity in our study, we analyzed only the group with DC-associated premolars, divided using the criteria from the study by Fuji et al., thus obtaining two subgroups of 38 and 9 patients. The study group of Qian et al. [24] comprised 11 patients diagnosed with 15 DC-associated mandibular premolars, where 15 healthy premolars in the other mandibular side served as the control group. Yahara et al. [25] evaluated 21 patients with 21 DC-associated mandibular premolars treated with marsupialization of the cysts. After surgery, the patients were divided into two subgroups: (1) those with teeth that erupted spontaneously (15 individuals) and (2) those with teeth that did not erupt during the 12-month follow-up after surgery (6 individuals).

For evaluating both the intervention and the comparison, most of the teeth erupted spontaneously after marsupialization. However, some required orthodontic traction (Table 2).

**Table 2:** Impact of variables defined in the methodology on teeth eruption after DC removal

	Fujii et al. [22]		Hyomoto et al. 1^23^]		Qian et al. [24]		Yahara et al. [25]	
Intervention and Comparison	Spontaneous eruption	Lack of spontaneous eruption	Spontaneous eruption	Lack of spontaneous eruption	Spontaneous eruption	Lack of physiological eruption of the opposite teeth	Spontaneous eruption	Lack of spontaneous eruption
Number of teeth/number of observations (n)	45/60	15/60	38/47	9/47	15/15	9/15	15/21	6/21 (no eruption after 360 days)
Time elapsed from marsupialization to orthodontic force application	X	NR	X	NR	X	No orthodontic treatment performed	X	NR
Eruption period (days)	90.4 ± 91.8	65.4 ± 141.5	109	NR	263 ± 33	807 ± 112	174 ± 84	NR
Mean age (years)	10.5 ± 1.9	13.7 ± 4.8	10.6 ± 2.0	13.1 ± 2.9	9.1 ± 0.8	9.1 ± 0.8	9.8 ± 2.1	13.2 ± 2.8
Sex (n)	33 M, 27F	34 M, 24F (whole group with canines)	7 M, 4F		12 M, 9F			
Cusp depth (mm)	4.5 ± 4.3	9.8 ± 5.7	4.4 ± 4.5	9.3 ± 6.31	5.4 ± 5.2	3.5 ± 2.7	S: 14, D:1^a^	S: 2, D: 4^a^
Angulation (°)	24.6 ± 21.5	52.7 ± 45.0	21.8 ± 20.4	67.7 ± 49.8	39.1 ± 31.6	10.5 ± 8.3	62.6 ± 19.6	26.6 ± 21.7
Root formation	NR	NR	< 1/2: 17, 1/2–3/4: 11, 3/4–4/4 (open apex): 9, mature root: 1	< 1/2: 3, 1/2–3/4: 0, 3/4–4/4 (open apex): 2, mature root: 4	< 1/2: 7, 1/2–3/4: 10	< 1/2: 6, 1/2–3/4: 7, 3/4–4/4: 4	< 1/2: 9, > 1/2: 6	< 1/2: 5, > 1/2:1
Cyst area (mm^2^)	513.0 ± 240.2	465.1 ± 344.2	533.8 ± 239.8	546.7 ± 406.5	231.9 ± 197.8	No cyst	NR	
Space/tooth size	1.1 ± 0.2	0.8 ± 0.6	1.0 ± 0.2	1.0 ± 0.5	1.26 ± 0.23	1.28 ± 0.24	1.08 ± 0.36	1.14 ± 0.12

*NR*, not reported; *M*, males; *F*, females

^a^S, D: shallower (S) or deeper (D) than the line that passes the one-half of the roots of the adjacent teeth

The results of the analysis of the impact of the variables defined in the methodology on tooth eruption after DC marsupialization are shown in Table 2. The risk of bias assessment is presented in Table 3, and the results of the qualitative assessment of articles based on QAS are given in Table 4.

**Table 3:** Risk of bias

Domain	Study			
	Fujii et al.	Hyomoto et al.	Qian et al.	Yahara et al.
Bias due to confounding	Serious risk	Serious risk	Serious risk	Serious risk
Bias in the selection of participants into the study	Critical risk	Critical risk	Critical risk	Critical risk
Bias in the classification of interventions	Serious risk	Serious risk	Serious risk	Serious risk
Bias due to deviations from intended interventions	Low risk	Low risk	Low risk	Low risk
Bias due to missing data	Low risk	Low risk	Low risk	Low risk
Bias in measurement of outcomes	Moderate risk	Moderate risk	Moderate risk	Moderate risk
Bias in the selection of the reported result	Moderate risk	Moderate risk	Moderate risk	Moderate risk
Overall	Critical risk	Critical risk	Critical risk	Critical risk

**Table 4:** Quality assessment according to the modified Newcastle—Ottawa Scale

Study	Selection (0–4 points)	Comparability (0–2 points)	Outcome assessment (0–3 points)
Fujii et al.	4	1	2
Hyomoto et al.	4	1	2
Qian et al.	4	2	3
Yahara et al.	4	1	3

The heterogeneity analysis showed substantial differences with respect to all parameters except the cyst area, so a fixed effects meta-analysis was performed for the cyst area and random effects meta-analyses were performed for the remaining parameters. The obtained results of the statistical analysis are presented in Table 5 and in Figs. 3, 4, 5, 6, 7 and 8.

**Table 5:** Heterogeneity analysis

Parameter	Q	I2
Percentage of spontaneously erupted teeth	7.55	0.60
Mean age	40.54	0.93
Cusp depth	17.78	0.89
Angulation	96.32	0.97
Root formation stage	4.78	0.58
Cyst area	0.48	0.00
Space/tooth size	12.18	0.75

**Fig. 2:**
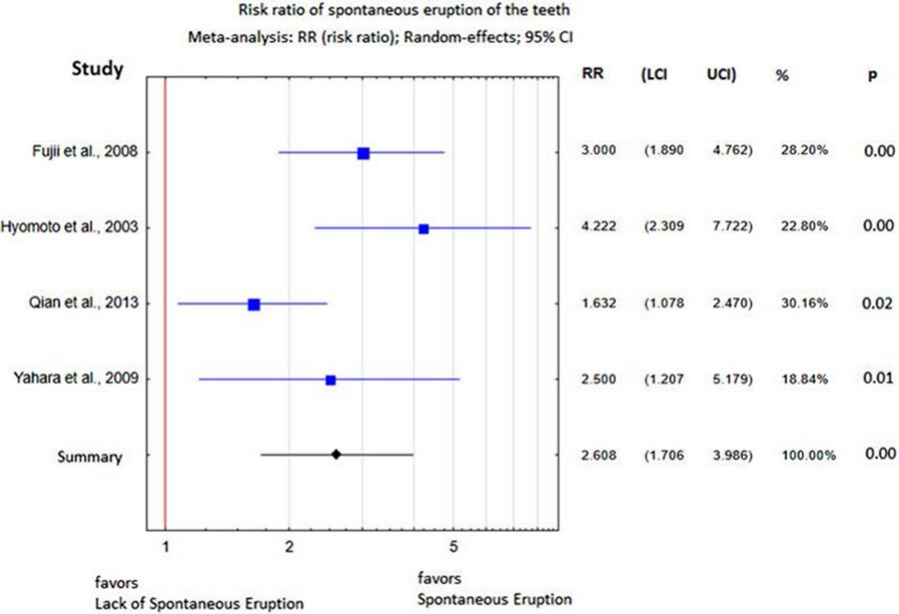
Risk ratio for spontaneous tooth eruption after marsupialization

The mean risk ratio for spontaneous eruption after marsupialization was 2.61, which means that nearly 62% of DC-associated premolars erupted spontaneously (Fig. 2), and it was statistically significant (*p* = 0.00) with a level of heterogeneity of I^2^ = 60%.

**Fig. 3:**
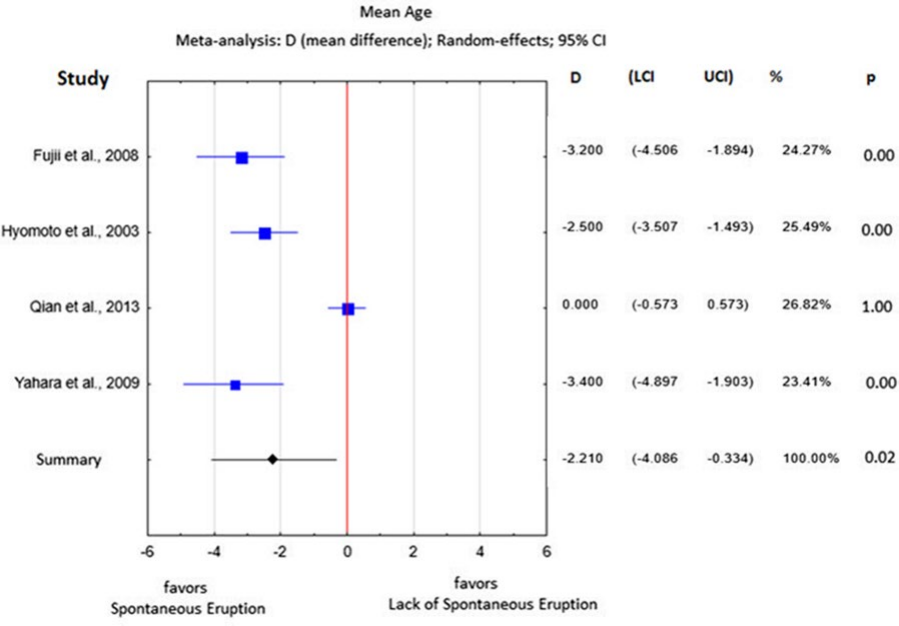
Age difference between the study group and the control group

The average age of patients experiencing spontaneous eruption in almost all studies was significantly lower than the average age of patients whose eruption process was arrested and was stated as 2.21 years on average (*p* = 0.02; Table 2; Fig. 3). The heterogeneity level was high (I^2^ = 93%).

**Fig. 4:**
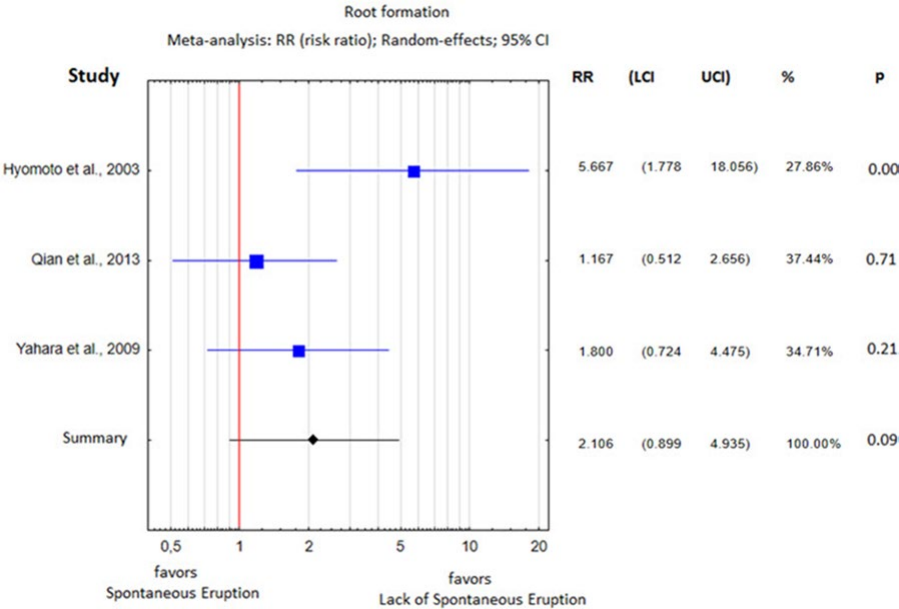
Root formation difference between the study group and the control group

Regarding the stage of root formation, defining the cutoff threshold as 1/2 of the fully developed root length, an average risk ratio of spontaneous eruption of 2.11 was determined (*p* = 0.09; Fig. 4). The meta-analysis presented a high degree of heterogeneity (I^2^ = 58%).

**Fig. 5:**
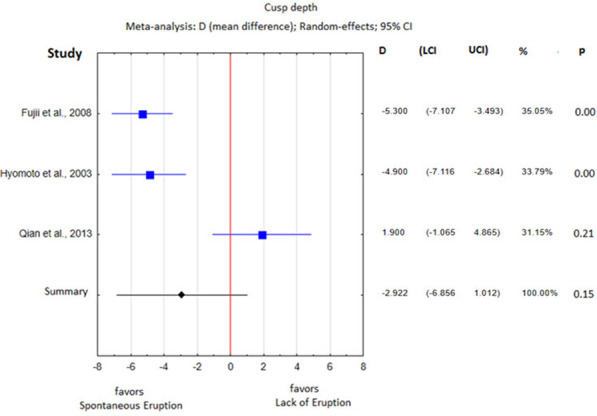
Cusp depth difference between the study group and the control group

The DC-associated premolar was located 2.92 mm shallower in the study group than in the control group; nevertheless, it was not significantly different (*p* = 0.15, Fig. 5), with a high level of heterogeneity (I^2^ = 89%).

**Fig. 6:**
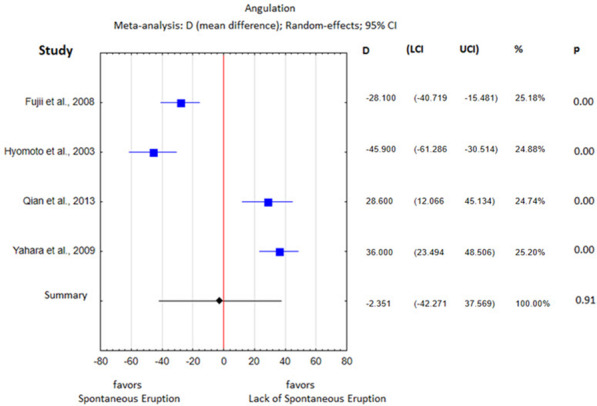
Tooth angulation difference between the study group and the control group

The angulation of the tooth did not have a statistically significant impact on spontaneous eruption (*p* = 0.91, Fig. 6). The level of heterogeneity was high (I^2^ = 97%).

**Fig. 7:**
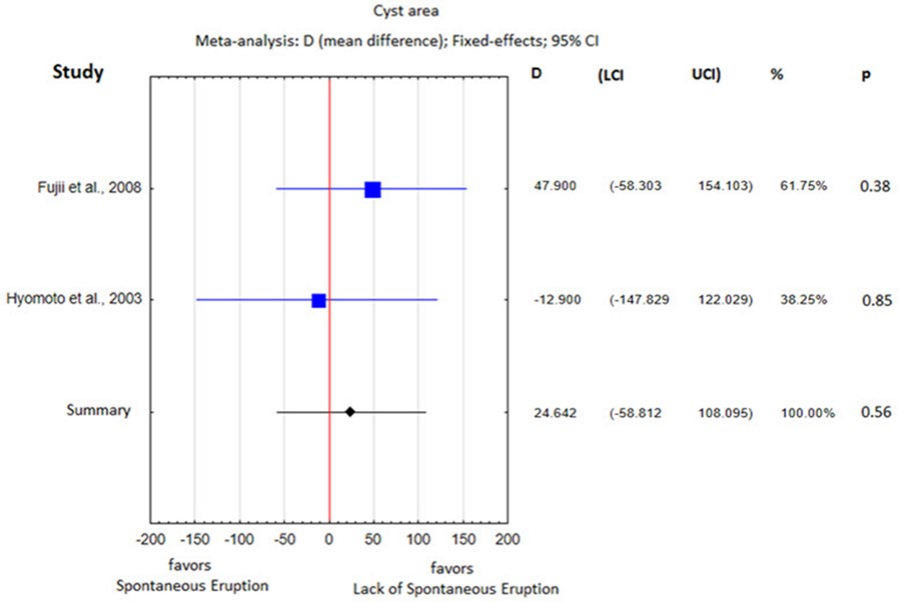
Cyst area difference between the study group and the control group

A meta-analysis conducted for the studies by Fujii et al. and Hyomoto et al. showed that the cyst area in the study group was slightly smaller than that in the control group, but the difference was not statistically significant (*p* = 0.56; Fig. 7). The existence of heterogeneity was not determined (I^2^ = 0%).

**Fig. 8:**
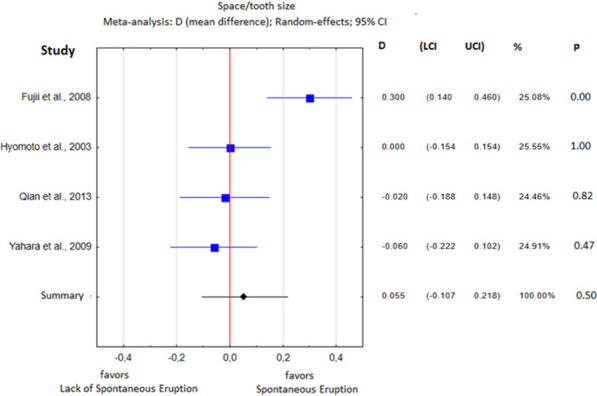
Eruption space/tooth size difference between the study group and the control group

No significant impact of the space/tooth ratio on spontaneous eruption was found (*p* = 0.50; Fig. 8). The level of heterogeneity was high (I^2^ = 75%).

The GRADE approach revealed very low-quality evidence from the individual studies (Table 6).

**Table 6:** GRADE summary of findings

Admission type	No. of studies	Study design	Risk of bias	Inconsistency	Indirectness	Imprecision	Other considerations	RR (95% CI)	Certainty
*Certainty assessment*									
RR (risk ratio) for spontaneous eruption of the teeth	4	Retrospective cohort studies	Serious	Serious^a^	Not serious	Serious^b^	None	2.608	Very low
D (mean difference) for mean age	4	Retrospective cohort studies	Serious	Serious^a^	Not serious	Serious^b^	None	NR	Very low
RR (risk ratio for root formation)	3	Retrospective cohort studies	Serious	Serious^a^	Not serious	Serious^b^	None	2.106	Very low
D (mean difference) for cusp depth	3	Retrospective cohort studies	Serious	Serious^a^	Not serious	Serious^b^	None	NR	Very low
D (mean difference) for angulation	4	Retrospective cohort studies	Serious	Serious^a^	Not serious	Serious^b^	None	NR	Very low
D (mean difference) for cyst area	2	Retrospective cohort studies	Serious	Serious^a^	Not serious	Serious^b^	None	NR	Very low
D (mean difference) for space/tooth size	4	Retrospective cohort studies	Serious	Serious^a^	Not serious	Serious^b^	None	NR	Very low

GRADE, Grading of Recommendations Assessment, Development, and Evaluation; RR, risk ratio; CI, confidence level; NR, not reported

^a^Different sample sizes and timings of studies lead to inconsistency in the results

^b^Very few studies published which raises the suspicion of publication bias

GRADE quality of evidence: High quality: We are very confident that the true effect lies close to that of the estimate of the effect. Moderate quality: We are moderately confident in the effect estimate: The true effect is likely to be close to the estimate of the effect, but there is a possibility that it is substantially different. Low quality: Our confidence in the effect estimate is limited. The true effect may be substantially different from the estimate of the effect. Very low quality: We have very little confidence in the effect estimate: The true effect is likely to be substantially different from the estimate of effect

## Discussion

Dentigerous cysts are an interdisciplinary problem. Their development is usually asymptomatic for a long time. A delay in tooth eruption, its absence in the dental arch or its incorrect position arouses anxiety. The consequences of DCs are as follows: (a) bone destruction; (b) root resorption; and (c) sometimes when untreated, transformation into an odontogenic tumor (e.g., ameloblastoma) or a malignant tumor (e.g., squamous cell carcinoma) [1, 2]. The need to evacuate the cyst is obvious. However, preservation of DC-associated teeth requires a reliable, intuitive protocol, which fully justifies the subject of our study.

Some authors claim that spontaneous tooth eruption should occur no later than three months after marsupialization; otherwise, orthodontic traction should be applied [7, 13, 19]. This is in accordance with the results of the study by Hyomoto et al. [23], where the teeth erupted within 109 days on average; after 110 days, the number of erupting teeth evidently decreased. The duration of spontaneous eruption was comparable or longer (5.8 ± 2.8 months) in the study by Yahara et al. [25]. Here, the authors determined that if the tooth did not erupt at least halfway within 3 months postsurgery, it would most likely not reach the occlusal plane even within 12 months. Fujii et al. [22] and Hyomoto et al. [23] observed a longer duration of spontaneous eruptions than forced eruptions... This difference, however, was either statistically nonsignificant [22] or not analyzed [23]. In the patients studied by Qian et al. [24], all teeth erupted spontaneously after marsupialization, in a significantly shorter time than the teeth without DC. This allowed the conclusion that marsupialization induces osteogenesis and pressure reduction in the alveolar bone, which may stimulate faster eruption. Nonetheless, such interpretation must be treated with caution; there are no reviews on the tooth eruption rate, which one can compare with the DC-associated tooth eruption duration time.

Spontaneous eruption occurred mostly in patients within the first decade and the beginning of the second decade of their life. The discussed difference was 2.21 years on average (*p* = 0.02; Table 2, Fig. 3). Only Qian et al. [24] did not show a statistically significant age difference between the study and control groups, which is evident since they compared DC-associated teeth with those from the other quadrant in the same mandible. The patient's age is closely related to the stage of root formation; therefore, these two factors may be analyzed simultaneously when assessing their impact on spontaneous tooth eruption after marsupialization. In the study by Hyomoto et al., an initial stage of root formation (r < 1/2) increased the probability of spontaneous tooth eruption after marsupialization in a statistically significant manner Nevertheless, other authors have not demonstrated such a relationship, indicating that eruption is utterly independent of the degree of root formation [24, 25]. Because the stages of root formation in the study and control groups differed from one article to another, the data are not entirely comparable. Nevertheless, we showed in the meta-analysis that when the developing root is shorter than 1/2 of its mature length, there is a doubled chance of spontaneous eruption of the DC-associated premolar.

It was impossible to determine the influence of patient sex on tooth eruption after marsupialization. The sex distribution in the study and control groups was not established in any of the studies.

Regarding the distance between the top of the tooth cusp and the edge of the alveolar process in a perpendicular projection, Fujii et al. [22] observed significantly lower values in cases of spontaneous eruption after marsupialization than in cases of cystectomy or application of orthodontic traction. On this basis, the authors concluded that the tooth's position at a depth of < 5.1 mm is one of the predictive parameters of the spontaneous eruption of a DC-associated tooth. Other authors have reached similar conclusions [23, 25]. Qian et al. [24] showed that despite the shallower location of the teeth in the control group (3.5 ± 2.7), the duration time of their eruption was more prolonged, although it was not a statistically significant difference. As for the angulation of the teeth that spontaneously erupted after marsupialization, in the studies of Fujii et al. [22] and Hyomoto et al. [23], it was two and three times smaller on average, compared to the angulation in cases requiring a cystectomy or orthodontic traction. On this basis, Fujii et al. [22] determined that angulation < 25° is one of the significant predictive parameters of DC-associated tooth eruption However, this conclusion was not confirmed in the works of Qian et al. [24] and Yahara et al. [25]; moreover, Qian et al. observed greater angulation values in the group of patients whose teeth erupted spontaneously. Such apparent contradiction among the studies was confirmed by the results of our meta-analysis. The area of a dentigerous cyst was not found to be a predictive factor of a spontaneous eruption. The average cyst area in the study groups, where spontaneous tooth eruption occurred after marsupialization, was similar in the studies by Fujii et al. [22] and Hyomoto et al. [23], while the study by Qian et al. [24] provided a much smaller value.. Based on our results, it can be concluded that from an orthodontic point of view, the discussed parameter is not a key parameter in treatment planning. However, it may have an impact on decisions regarding the surgical procedure.

For the space/tooth size ratio, Fujii et al. [22] described a statistically significant difference between groups, therefore concluding that a ratio greater than 1:1 is a factor conducive to spontaneous eruption). In the remaining studies, no statistically significant differences were found, which influenced the results of our meta-analysis. The risks of bias in the pre-intervention domain were considered serious in all works. The reasons were as follows: (1) the failure to use methods to account for confounders, such as stratification, regression, matching, standardization or inverse probability weighting; (2) the retrospective nature of the research, namely, forming study and control groups after the results have been achieved (thus, prognostic variables might have been deliberately selected to confirm the assumed hypotheses); and (3) the selection of participants from a larger group of patients treated in a given unit in a specific timeline, which increases the probability of disturbing the proportion of a primary cohort. This study did not adopt a critical risk because the study group's inclusion criteria were precisely defined in all studies.

Since there was no misclassification of the intervention status, the intervention domain's risk was assessed as low. The study participants were routinely treated using standard procedures, and medical records were maintained. Ongoing records were entirely available to the authors; therefore, the risks of bias due to deviations from the intended interventions and due to missing data were considered low. The risks of bias in the measurement of outcomes were defined as moderate because the methods of assessing the results were objective and carried out in the same way for the study and control groups. Nevertheless, the lack of randomization made the measurement results potentially dependent on whether the patient belonged to the study or control group.

The risk of bias in selecting the reported results was also moderate because the authors provided all the results without using the data obtained in previous analyses, despite the studies' retrospective nature.

The risk of bias in all four studies was considered critical. However, despite serious risks of bias of all qualified works, the serious risks were recorded only in the pre-intervention domain. The reason was the retrospective nature of the study, which is an obvious disadvantage; however, this is easy to overcome in future studies on this aspect.

For the qualitative assessment based on QAS, in the "Patient Selection" category, the works by Fujii et al. [22], Hyomoto et al. [23], and Yahara et al. [25] obtained 4 points because the patients were appropriately classified into groups according to a differentiating factor, which was either spontaneous eruption or the lack thereof after marsupialization. Moreover, full documentation was available, showing that the studied effects did not occur at the beginning of the research. The article by Qian et al. [24] lost 0.5 points for low representativeness of the study group and another 0.5 points due to the qualitatively weaker control group, which were the participants' healthy teeth from the study group. In turn, the construction of study and control groups by Qian et al. provided no additional differentiating factors. Therefore, this paper awarded 2 points for the section of confounding factors. The articles by Fujii et al. [22] and Hyomoto et al. [23] lost 1 point due to the age differences between the control and study groups, while the work of Yahara et al. [25] did not receive points in this category due to significant differences in the size of the control and study groups, which could be a significant factor affecting the studied variables. In the effect assessment section, all articles lost 1 point each due to the lack of evaluator blinding. The works of Fujii et al. [22] and Hyomoto et al. [23] lost 1 additional point because orthodontic traction was applied sooner than three months after marsupialization or cystectomy, which could have influenced the assessment of time needed for a tooth to erupt spontaneously.

The meta-analysis of selected articles showed that nearly 62% of DC-associated premolars erupted spontaneously after cyst marsupialization/decompression. The meta-analysis also found statistically significant relationships between patient age and root formation stage and the probability of spontaneous eruption. It should be emphasized, however, that the limited number of works qualified for inclusion in the meta-analysis could significantly distort the results.

The level of heterogeneity was high in all analyzed studies. Nevertheless, the I^2^ value was significantly lower in the following discussed parameters: percentage of spontaneously erupted teeth, root formation stage and cyst area. Therefore, the conclusions drawn based on these results might be more reliable than the rest.

The assessment of the certainty of evidence revealed the low quality of the articles. The inconsistency was considered serious due to different sample sizes and timings of the individual studies. Moreover, a small number of published studies raised the suspicion of publication bias, and therefore, the imprecision was assessed seriously. Taking into account the serious risk of bias in all qualified studies, we assessed the certainty of evidence as very low for all the analyzed factors.

### Limitations

In this systematic review, the most prominent databases were searched using a comprehensive search strategy. However, non-English searches were not undertaken, posing a risk of incomplete yield, which could have had an impact on the obtained results due to publication bias.

To date, very little research has been conducted on the management of DC-associated teeth. Except for merely four retrospective works, which were selected, only case reports and case series are available [9–21, 26, 27], where the authors present selected methods of treatment based on individual clinical experiences or protocols adopted in a given unit, which is not in line with evidence-based medicine. The high heterogeneity and small number of the analyzed papers resulted in a very low certainty of evidence. It is therefore necessary to plan and conduct similar research (especially RCTs and CCTs) on large groups in order to be able to draw clear conclusions.

## Conclusion

The small number of published studies, as well as differences among the available studies in terms of the size of the groups and their characteristics such as age, sex, and the period of observation, do not allow us to provide a list of objective criteria allowing for decisions about the implementation or withdrawal of orthodontic traction after marsupialization. Young age (approximately 10 years) and the root formation stage of the tooth below half of its total length seem to be factors that increase the probability of a spontaneous eruption. Determining the influence of the remaining factors requires further research, primarily with the same inclusion criteria and homogeneous groups, which would enable the development of a reliable and reproducible protocol for the management of DC-associated teeth, which is much needed in everyday clinical practice.

## Supplementary Information

**Additional file 1:** Search strategy.

## Data Availability

The datasets used and/or analyzed during the current study are available from the corresponding author on reasonable request.

## Abbreviations

DC: Dentigerous cyst
PRISMA: Preferred reporting items for systematic reviews and meta-analyses
PICO: Population, intervention, comparison, outcome
RCT: Randomized clinical trial
CCT: Controlled clinical trial
ROBINS-I: Risk of bias in non-randomized studies of interventions
QAS: Quality Assessment Scale
GRADE: Grading of recommendations assessment, development, and evaluation
