# Supplementary material for: Factors influencing an eruption of teeth associated with a dentigerous cyst: a systematic review and meta-analysis
Source: BMC Oral Health. 2021 Apr 7;21:180. doi: 10.1186/s12903-021-01542-y (PMC8028237; doi:10.1186/s12903-021-01542-y)
Supplement: Supplementary file 1 — Additional file 1: Search strategy. [file 12903_2021_1542_MOESM1_ESM.docx]

**Supplement 1**

**Search strategy**

| **Database** | **Keywords** | **Limits** |
| --- | --- | --- |
| PubMed | - (((Teeth) AND (Dentigerous cyst)) OR (Odontogenic cyst)) OR (Follicular cyst) - (((Orthodontics) AND (Dentigerous cyst) OR (Odontogenic cyst) OR (Follicular cyst) - (((((Human teeth) AND (Eruption pattern) OR (Eruption timing) OR (Eruption interval) OR (Eruption period) OR (Eruption duration) | Randomized clinical trials (RCT), prospective controlled clinical trials (CCT), case series, observational studies, review articles, and retrospective studies, English language, studies on humans |
| EMBASE | - (((Teeth) AND (Dentigerous cyst)) OR (Odontogenic cyst)) OR (Follicular cyst) - (((Orthodontics) AND (Dentigerous cyst) OR (Odontogenic cyst) OR (Follicular cyst) - (((((Human teeth) AND (Eruption pattern) OR (Eruption timing) OR (Eruption interval) OR (Eruption period) OR (Eruption duration) | Randomized clinical trials (RCT), prospective controlled clinical trials (CCT), case series, observational studies, review articles, and retrospective studies, English language, studies on humans |
| Cochrane Central Register of Controlled Trials | - (((Teeth) AND (Dentigerous cyst)) OR (Odontogenic cyst)) OR (Follicular cyst) - (((Orthodontics) AND (Dentigerous cyst) OR (Odontogenic cyst) OR (Follicular cyst) - (((((Human teeth) AND (Eruption pattern) OR (Eruption timing) OR (Eruption interval) OR (Eruption period) OR (Eruption duration) | Randomized clinical trials (RCT), prospective controlled clinical trials (CCT), case series, observational studies, review articles, and retrospective studies, English language, studies on humans |
